# Nanolime dispersions as active agents in self-healing lime mortars for durable heritage preservation

**DOI:** 10.1617/s11527-026-03007-6

**Published:** 2026-03-02

**Authors:** C. De Nardi, J. Annear, E. Gualini, S. Morrocchesi, R. Giorgi

**Affiliations:** 1 School of Engineering, Cardiff University, Queen’s Buildings, The Parade, Cardiff, CF243AA Wales, UK; 2 Consorzio per lo Sviluppo dei sistemi a Grande Interfase (CSGI), University of Florence, via della Lastruccia 3, 50019 Sesto Fiorentino, Italy

**Keywords:** Self-healing, Lime-based mortars, Nanolime dispersions, Built heritage

## Abstract

Built heritage is facing growing exposure to diverse and severe risks as the impacts of climate change intensify. To address these challenges, historic masonry repair techniques must evolve to support more resilient and durable preservation strategies. In this context, recent advances in self-healing concrete technologies offer promising pathways to adapt and transfer these approaches to heritage conservation, particularly for lime-based materials used in historic masonry. This study investigates the potential of nanolime dispersions as self-healing agents for natural hydraulic lime (NHL) mortars, applied through vascular network systems specifically designed to be fully integrated within mortar joints while ensuring chemo-physical and mechanical compatibility with the host material. Two water–ethanol formulations (50:50 and 80:20 ratios) were evaluated for their impact on mechanical performance and physicochemical compatibility with NHL matrices. Healing efficacy and compatibility were examined using three-point bending tests, FT-IR, XRD, and SEM analyses. Although no autogenous healing was observed, the nanolime treatments achieved up to 12% strength recovery, while stiffness recovery remained negligible. Notably, this nanolime-induced healing effect persisted for up to 280 days, highlighting its long-term potential. FT-IR and XRD results indicated possible formation of C-S-H phases, and SEM imaging confirmed morphological compatibility with the NHL matrix. Overall, the findings support the application of nanolime dispersions as a viable agent of self-healing strategies for the durable and compatible repair of lime-based heritage mortars.

## Introduction

Historic buildings withstood significant climatic events, demonstrating resilience against earthquakes, accidents, cycles of moisture wetting–drying, and freezing [1]. Nevertheless, climate change is increasing the frequency and severity of deterioration mechanisms, placing renewed stress on historic masonry systems and highlighting the need for compatible and durable repair strategies. In this context, lime-based mortars, widely used in historic construction, play a crucial role in accommodating deformation and protecting masonry units, but they remain vulnerable to cracking and progressive damage over time [2].

Evidence from historic structures indicates that lime-based mortars can exhibit a natural capacity for crack sealing and microstructural repair. Moropoulou et al. [3] reported the formation of secondary calcitic materials within cracks of Byzantine monuments dating from the eleventh–thirteenth centuries, suggesting self-healing processes activated by seismic damage. Comparable phenomena were reported in historic lime mortars from the Chellah Mosque in Morocco, where petrographic analyses identified secondary calcite precipitation within microcracks, associated with local porosity reduction and partial crack infilling [4]. These findings demonstrate that lime mortars can undergo self-healing phenomena under favourable environmental conditions [4].

Similar to concrete, lime-based mortars possess a so-called autogenous self-healing ability, defined as a natural process of filling and sealing cracks without any external operations and works [5]. This mechanism determines the mechanical properties recovery and enhances performance after damage [6].

Early work by Anderegg showed that infiltrating water can dissolve calcium carbonate and hydroxide phases, transport them through the pore network and promote reprecipitation within cracks [7]. Subsequent studies highlighted the importance of moisture availability, carbonation gradients, and partially carbonated lime particles in driving these processes [8]. Partially carbonated particles can dissolve, be transported, and later reprecipitate elsewhere. Additionally, impurities such as Na(OH) and Na₂CO₃ may enhance calcite solubility, further promoting the healing process [9]. Experimental investigations on natural hydraulic lime (NHL) mortars have shown that autogenous healing can lead to partial mechanical recovery, with strength regain ranging between 20 and 40%, depending on crack width, curing age, and damage level [10–12]. However, this mechanism is strongly limited by crack size, material maturity, and environmental exposure, and becomes increasingly ineffective for wider cracks or older mortars [11].

Recently, to overcome the intrinsic limitations of autogenous healing, engineered self-healing strategies have been extensively developed for cementitious materials [6, 13]. In particular, in concrete, a wide range of engineered self-healing technologies has been proposed to mitigate cracking and related deterioration processes, thereby restricting the ingress of chemically aggressive agents and moisture that accelerate damage, while also contributing to reduced material consumption through extended service life and fewer repair interventions [14–18]. These approaches are commonly classified as intrinsic, capsule-based [16, 19, 20], or vascular-based systems [6, 13]. Among engineered self-healing strategies, vascular-based systems offer a distinctive advantage by enabling repeated or on-demand delivery of healing agents, overcoming the single-use limitation typical of intrinsic and capsule-based approaches [18]. Since the early work by Dry [21] using embedded glass tubes, vascular networks have evolved through a range of manufacturing solutions, including hollow tubes, sacrificial elements, and more recently 3D-printed networks, each presenting specific advantages and limitations in terms of integration, durability, and flow reliability [22–27]. Hollow and rigid inclusions have been reported to introduce stress concentrations and practical issues such as clogging [18, 28], while complex 3D-printed vascular networks may involve higher manufacturing costs and implementation challenges [23, 29, 30], particularly in contexts requiring material compatibility and adaptability. Sacrificial vascular systems, in which temporary inclusions are removed after setting to create interconnected channels, represent a pragmatic compromise, enabling effective healing-agent delivery while minimising mechanical incompatibility with the host matrix [30–32]. A robust implementation of this approach was developed at Cardiff University, where two-dimensional vascular networks are formed by removing embedded polyurethane tubes from cementitious matrices [31–34]. The effectiveness and scalability of this system have been demonstrated through full-scale site trials, confirming reliable agent delivery and structural integration under realistic conditions [34].

Current engineered self-healing strategies are largely developed for Portland cement systems and often rely on materials that are incompatible with the chemical, mechanical, and aesthetic requirements of historic masonry [35]. Consequently, a significant knowledge gap exists in developing repeatable, compatible self-healing solutions specifically for NHL-based restoration mortars [24].

Mortars used in the restoration of historic buildings must be carefully formulated to enhance resilience and ensure long-term durability, especially in response to climate change-related challenges. In this light, researchers are exploring various approaches, such as bio-based healing agents [36, 37], mortar mini vascular networks [38, 39] and autonomous crack repair mechanisms [11, 12] to enhance the durability of historic masonry without compromising its authenticity. These healing mechanisms typically operate only once, as the healing agents are exhausted after their initial activation.

However, the development of systems capable of multiple healing cycles, such as vascular networks, would offer significant advantages, particularly in the context of built heritage conservation. Ideally, vascular networks should enable the repeated or on-demand delivery of healing agents over time, supplying them directly to damaged zones as deterioration occurs. The selection of an appropriate healing agent is therefore critical to the success of such systems. It must exhibit long-term stability and actively promote the self-healing process within the mortar matrix. Key parameters include low viscosity, to ensure efficient flow through the vascular channels and effective crack penetration, chemical compatibility with historic materials, and non-toxicity to support environmental sustainability and safe application in conservation contexts.

Bio-stimulated and bacteria-based self-healing strategies are emerging for lime-based mortars, but current applications remain largely at laboratory or small pilot scale and are far less mature than the extensive developments in self-healing concretes and cementitious repair mortars [40–44]. Only a limited number of studies have so far examined bacterial or nanolime bio-additives specifically tailored for historic lime mortars and masonry substrates, where microbially induced mineral precipitation can promote crack sealing, yet performance remains strongly dependent on moisture, temperature, and nutrient availability, making control and repeatability particularly challenging under real heritage exposure conditions [36, 45, 46]. Silicate-based treatments, such as sodium silicates, have been used in self-healing and densification strategies for concrete [13, 38, 47–50] but the introduction of mobile alkali ions can promote salt migration, efflorescence, and surface alteration, raising compatibility concerns for historic materials [51, 52] Lithium silicates, characterised by lower alkali mobility and reduced salt formation, are therefore considered more compatible [53, 54] and although typically applied as surface treatments, they have been proposed as candidate healing agents within vascular delivery concepts for lime-based mortars; however, their long-term chemical and aesthetic compatibility is still under investigation [55]. By contrast, many polymeric healing agents developed for Portland-cement systems [33, 56] may achieve more pronounced crack closure, but their long-term ageing, reversibility, vapour permeability, and chemo-mechanical compatibility can be problematic when transferred to lime-based repair mortars for heritage conservation.

In that perspective, ongoing research by the author aims to design vascular networks that can be fully integrated into mortar joints while ensuring chemo-physical and mechanical compatibility.

Criteria for the selection of effective healing agents include: (i) maintaining a liquid state and chemical stability over extended periods, (ii) reacting with the lime-based matrix upon release to form compatible and durable compounds, and (iii) enhancing and complementing the natural autogenous healing capacity of natural hydraulic lime.

This study has three main objectives: first, to evaluate the healing performance of nanolime dispersions when applied through sacrificial channels forming a vascular network, in order to isolate their specific contribution from the vascular network manufacturing process (e.g. 3D printed channels); second, to assess the healing efficacy over time and investigate the reactivity of the nanolime within the lime-based matrix; and third, to examine the chemo-physical compatibility of the NHL-nanolime system, a critical requirement for applications in built heritage conservation. To this end, a well-established vascular network manufacturing approach and standardised healing evaluation methods were adopted, ensuring controlled conditions for assessing nanolime performance without interference from external variables (as described in Sect. 2.1 Essential background). Two nanolime dispersions were tested, namely 50:50 = 10 g/L calcium hydroxide in 50–50% water–ethanol and 80: 20 = 10 g/L calcium hydroxide in 80–20% water- ethanol [57]. First, the degree of wetting was assessed through contact angle measurements. Subsequently, the effectiveness of the healing system was evaluated based on the recovery of mechanical strength and stiffness in samples subjected to three-point bending tests.

In summary, despite extensive research on self-healing systems for cementitious materials, effective solutions tailored to natural hydraulic lime mortars and compatible with historic masonry remain limited. Recent studies indicate that nanolime dispersions combine chemical compatibility, high reactivity, and favourable flow and penetration in porous substrates [58, 59]. Accordingly, this study hypothesises that suitably formulated nanolime can be transported through sacrificial vascular networks and react effectively within NHL mortars to promote crack stabilisation and mechanical recovery while preserving heritage compatibility. The original contribution lies in isolating and quantifying the performance of nanolime as a vascular-delivered healing agent and assessing its chemo-physical suitability for self-healing in conservation mortars. The paper investigates cracks of 0.05 and 0.1 mm, a range spanning incipient microcracks to hairline cracks in lime-based masonry joints, i.e. the earliest stage of discrete cracking that precedes visible macrocrack formation [60, 61]. These narrow openings are sufficient to disrupt the continuity of the mortar joint and reduce its effective stiffness, as shown in damage-mechanics-based models and micromechanical analyses of cracking localised in masonry joints [61, 62]. Microcracks and voids within bedding and pointing mortars are also known to exert primary control over moisture penetration and capillary flow in deteriorated masonry [63, 64]. Such small cracks can therefore act as early preferential pathways for transport-driven degradation and as precursors to the damage states recognised in masonry damage classifications, long before wider cracks that govern serviceability or stability are reached [65, 66]. In this regime, crack openings on the order of 102 µm are expected to modify the effective stiffness and continuity of the mortar joint, while corresponding to crack-width ranges commonly reported as governing fluid ingress, vapour transport, and serviceability thresholds, and serving as precursors to structural damage states, in lime mortars and historic masonry systems [60, 64, 65, 67].

The paper is structured as follows:

- Section 2 describes the materials and methods used in the study; including the experimental programme, detailing the procedures and testing arrangements applied to the raw materials, healing agent formulations, and the vascular network system embedded within lime-based mortar samples
- Section 3 presents the experimental results, discusses their relevance, and explores potential directions for future research.
- Section 4 summarises the study’s conclusions.

## Materials and methods

### Essential background

**Fig. 1:**
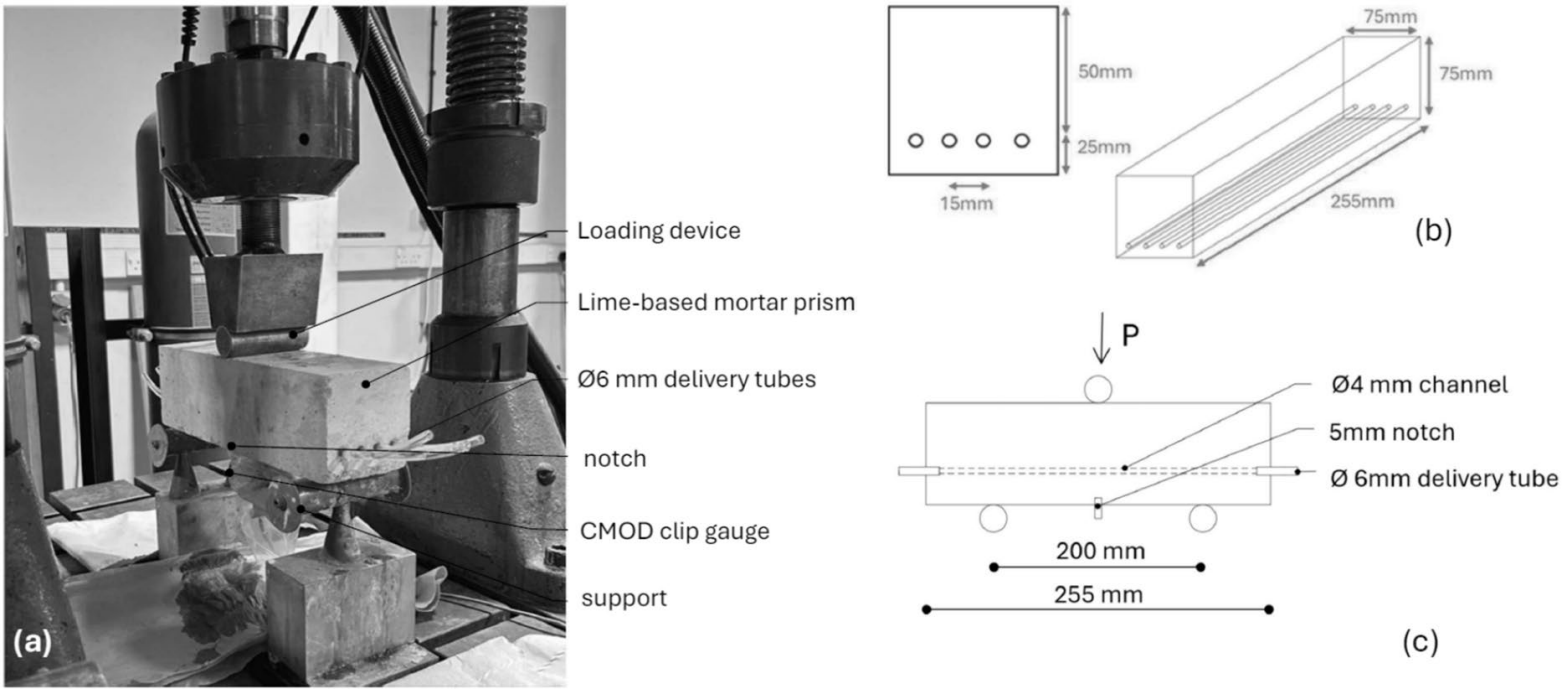
Annotated photograph of the beam specimen in the testing rig (**a**); samples dimensions (**b**), test arrangement (**c**)

To analyse the effectiveness of nanolime dispersions as healing agents, a well-established method for creating vascular networks in cementitious materials was adapted. Sacrificial channels, based on the approach developed by Davies et al. [31, 33, 68] were embedded into prismatic samples to enable direct interaction between the healing agent and the binder matrix. This technique was deliberately chosen over 3D-printed encapsulation systems to minimise extraneous physico-chemical influences and allow a clearer assessment of the nanolime's contribution to mechanical recovery and material compatibility. Control specimens incorporating identical vascular networks with empty channels, together with a deliberately simplified and uniform channel geometry, were employed to minimise geometric and mechanical interference effects and to decouple the delivery role of the vascular system from the chemical contribution of the nanolime dispersions, thereby isolating the specific role of the healing agent in the observed response. For clarity, essential information on the vascular manufacturing process and test setup (Fig. 1), is recapped here. Each mould featured pre-drilled stop-ends, and the vascular network was formed using 4 mm diameter PET tubes embedded within prismatic moulds (75 × 75 × 255 mm, Fig. 1b). The tubes were tensioned by hand, secured with crocodile clips on the external faces of the moulds during casting, and subsequently removed after 7 days of curing. Following demoulding, specimens were prepared with a 5 mm central notch on the lower surface to ensure controlled crack initiation during three-point bending tests (as represented in Fig. 1c). The channel openings were then widened and fitted with PET supply tubes to allow injection of the healing formulation (Fig. 1c). Controlled damage was introduced using three-point bending tests with a pre-formed notch to ensure cracking at mid-span (Fig. 1a). Cracks were induced up to defined crack mouth opening displacement (CMOD) thresholds (0.05 mm and 0.1 mm), reflecting the typical range found in historic lime masonry. The three-point bending tests were carried out using a Servocon System machine equipped with a 100 kN load cell, with a CMOD rate of 0.0001 mm/s, which was maintained throughout all loading and reloading stages. Healing performance was assessed using a three-stage process: (1) Pre-cracking: loading to failure or to specified CMOD; (2) Healing: 14-day curing under lab conditions (20 ± 5 °C, 45% RH); (3) Post-healing: reloading to failure.

Healing efficiencies can be determined by comparing pre-cracking and post-healing responses to those of control specimens. As indicated by Homma et al. [69] and commonly adopted by other investigators [23, 33, 38, 70, 71] the healing (or recovery) index can be calculated using the following equation:

1 $$ \eta_{\chi }^{k} \left( \% \right) = \frac{{\overline{\chi_{{{\mathrm{healed}}}}^{k}} - \overline{\chi_{{{\mathrm{damaged}}}}^{k}} }}{{\overline{\chi_{{{\mathrm{undamaged}}}}^{k}} - \overline{\chi_{{{\mathrm{damaged}}}}^{k} }}} \times 100 $$

where:

$\chi_{{{\mathrm{healed}}}}^{k}$ is the recovered property (e.g., strength σ or stiffness K) after the healing period (stage 3);

$\chi_{{{\mathrm{damaged}}}}^{k}$ is the residual value measured after the prescribed pre-damage condition (stage 2);

$\chi_{{{\mathrm{undamaged}}}}^{k}$ represents the reference value obtained from the same specimens in the undamaged state (stage 1).

So that: $\eta_{\sigma }^{k}$ indicates the healing index in terms of strength; $\eta_{K}^{k}$ indicates the healing index in terms of stiffness.

Detailed descriptions of the casting process, the formation of the vascular system, and the setup are also provided in [31, 33, 68].

#### Mix details

The prismatic samples were prepared using NHL 3.5 (natural hydraulic lime made from limestone with clay, fired at low temperatures (under 1250 °C), supplied by Tarmac in accordance with EN 459-1 [72] and fine aggregate sand with a particle size of 0–2 mm, in line with BS EN 13139:2013 [73]. The binder-to-sand ratio was kept at 1:3 by weight, and the water-to-lime ratio at 1:0.7, as is common practice for historical mortars [74–76]. The mixing protocol is detailed in De Nardi et al. [57]. Prisms, as shown in Fig. 1b, were cast in three layers, with the first 10 mm-thick layer serving as a bed the channels. After casting, the samples were left in the moulds, covered with a damp hessian sheet, for seven days. After demoulding, they were stored under laboratory environmental conditions (20 °C ± 5, RH ~ 45%) until the first test.

### Mock-up samples for material characterisation

**Fig. 2:**
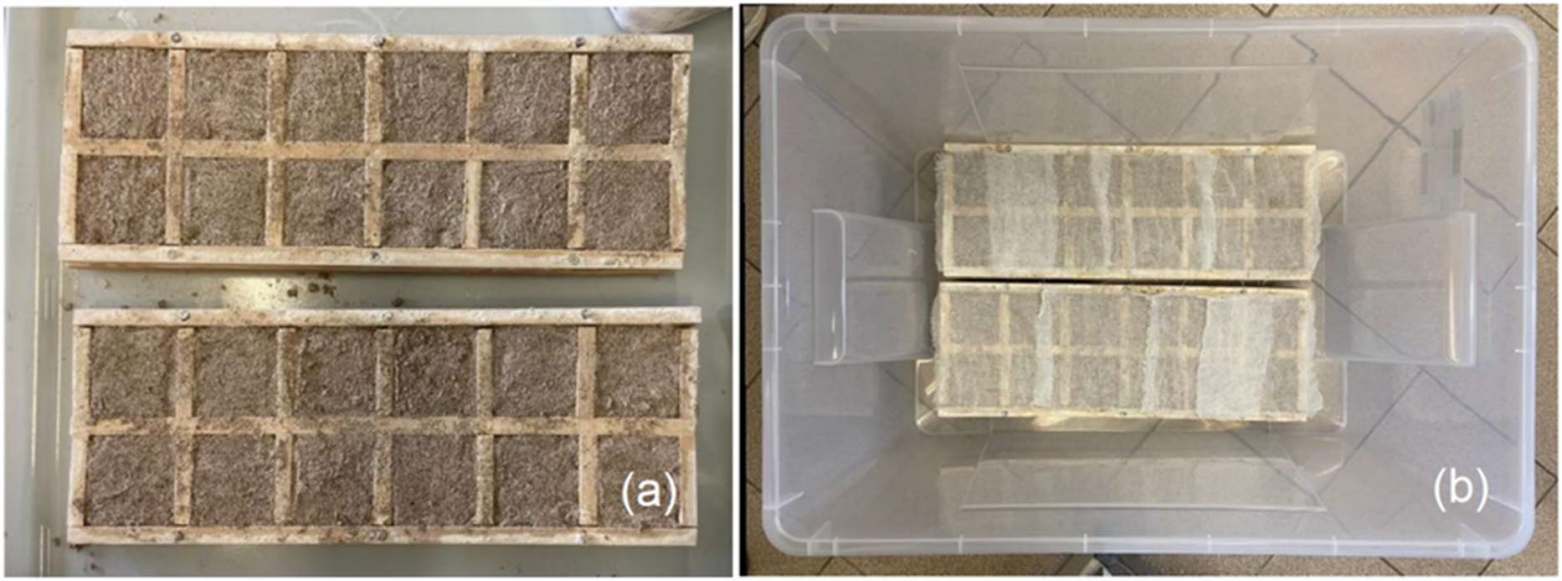
Mock-up samples after casting (**a**); samples enclosed in a sealed transparent container for curing under controlled laboratory conditions (**b**)

Fourier Transform Infrared Spectroscopy (FT-IR) (in both Attenuated Total Reflection (ATR) and transmittance modes) and X-Ray Diffraction (XRD) analyses were used to characterise the chemical and mineralogical composition of the raw materials (sand and hydraulic lime NHL 3.5), assess mock-up samples over time, and evaluate the impact of nanolime treatments. Mock-up samples were prepared using the same raw materials and mixing ratios as those used in the prismatic samples (as described in Sect. 2.1.1). The hydraulic lime mortar samples were cast into 55 × 55 × 10 mm wood moulds and covered with a damp cloth for 7 days to ensure proper curing, as represented in Fig. 2.

### Healing agents

Tailored nanolime dispersions were prepared by CSGI (University of Florence), specifically: a 50:50 formulation containing 10 g/L calcium hydroxide in a 50–50% water—ethanol mixture, and an 80:20 formulation with the same calcium hydroxide concentration in an 80–20% water–ethanol mixture. The calcium hydroxide consisted of laboratory-synthesised colloidal nanoparticles, produced by a solvothermal route, with platelet-like morphology and a mean particle size in the range of tens to a few hundreds of nanometres, as reported in [77, 78]

Compared to commercial products, the trialled dispersions were optimised for compatibility with vascular self-healing systems. The use of water–ethanol mixtures was adopted to balance colloidal stability, viscosity, and transport through micro-scale channels: ethanol reduces particle agglomeration, improves dispersion stability, and lowers surface tension, thereby enhancing penetration and flow in fine cracks and porous lime matrices, whereas purely aqueous systems exhibit faster aggregation and reduced injectability [57]. Importantly, the alcohol content was deliberately minimised, particularly in the 80:20 formulation, as a step toward more sustainable and environmentally friendly solutions. Reducing ethanol content lowers volatile organic compound (VOC) emissions, decreases flammability and toxicity risks during application, and improves overall safety for both operators and heritage environments. The chemical and colloidal stability of such nanolime blends has already been characterised and validated in previous work [57], confirming their suitability for long-term storage and effective dispersion performance.

### Methods

#### Analysis of wettability

Dynamic contact angles (SCA) values were recorded for the duration of 10 s, using a contact angle goniometer (DataPhysics OCA100, Germany) together with a camera-based optical measurement system at a room temperature (i.e. 20 ± 5 °C and a relative humidity around 50%). Glass microscope slides (22 × 50 × 0.15 mm^3^, Deckglasser, Menzel-Gläser, Germany) were employed as substrates, providing a smooth, chemically inert, and reproducible surface suitable for evaluating the intrinsic wettability of the nanolime dispersions. This approach was adopted to avoid the rapid liquid uptake associated with porous substrates, such as natural hydraulic lime mortars, which would compromise the accurate and consistent determination of the initial wetting behaviour.

The shape of a 0.5 μL droplet of a testing liquid deposited on the measured surfaces was captured by the camera once the equilibrium state was reached. The SCA was then determined by analysing the image using a software (SCA20) provided by DataPhysics.

#### Mechanical tests

Two curing stages were considered: an early stage (14 and 28 days) and a medium stage (280 days). After curing, the prisms were subjected to a three-point bending test in accordance with BS EN 12390-5 [79], and loaded either until failure (control series) or until a prescribed CMOD was reached, followed by unloading. The test was conducted using a CMOD rate of 0.0001 mm/s.

#### Experimental programme

The healing performance was analysed using prismatic samples, according to the experimental programme outlined in Table 1, which is organised into three main groups. The sample designation is defined as follows: A_B_C_D_E where A is the group number; B indicates the presence and number of the channels (0 indicates plain lime; 2 indicates 2 channels, 4 indicates 4 channels); C represents the age of the first test in days; D indicates the healing agent used to fill the channels (50:50 = 10 g/L calcium hydroxide in 50–50% water–ethanol; 80: 20 = 10 g/L calcium hydroxide in 80–20% water–ethanol, E is the level of the damage, considered in terms of crack width (CMOD equal to 0.01 mm = 0.01, CMOD equal to 0.05 mm = 0.05). Three prisms were cast for each sample designation.

**Table 1:** Experimental programme for mechanical testing

	Sample designation	No channels			Age of first test (days)			Healing agents		Crack width CMOD (mm)		Healing period (14 days)	FT-IR and XRD
		0	2	4	14	28	280	80:20	50:50	0.1	0.05		
GROUP 1	1_0_14	✓			✓			–	–	–	–	–	
	1_4_14_0.1			✓	✓			–	–	✓	–	✓	
	1_2_14_80:20_0.1		✓		✓			✓	–	✓	–	✓	
	1_2_14_50:50_0.1		✓		✓			–	✓	✓	–	✓	
	1_4_14_80:20_0.1			✓	✓			✓	–	✓	–	✓	
	1_4_14_50:50_0.1			✓	✓			–	✓	✓	–	✓	
GROUP 2	2_0_28	✓				✓		–	–	–	–	–	
	2_2_28_0.1		✓			✓		–	–	✓	–	✓	
	2_4_28_0.1			✓		✓		–	–	✓	–	✓	
	2_2_28_80:20_0.1		✓			✓		✓	–	✓	–	✓	
	2_2_28_50:50_0.1		✓			✓		–	✓	✓	–	✓	
	2_4_28_80:20_0.1		✓	✓		✓		✓	–	✓	–	✓	
	2_4_28_50:50_0.1		✓	✓		✓		–	✓	✓	–	✓	
GROUP 3	3_4_28_0.05			✓		✓		–	–	–	✓	✓	
	3_4_28_80:20_0.05			✓		✓		✓	–	–	✓	✓	
	3_4_28_50:50_0.05			✓		✓		–	✓	–	✓	✓	
	3_4_280_0.05			✓			✓	–	–	–	✓	✓	✓*
	3_4_280_80:20_0.05			✓			✓	✓	–	–	✓	✓	✓*
	3_4_280_50:50_0.05			✓			✓	–	✓	–	✓	✓	✓*

^*^Samples collected and analysed as per Table 2

Group 1 had two main objectives: first, to evaluate the influence of incorporating either two or four vascular channels on the flexural strength of early-age (14-day cured) lime-based prisms; and second, to assess both autogenous healing (in samples with empty channels) and engineered healing performance, activated by the two nanolime dispersions (named as 80:20 and 50:50) delivered in varying volumes (i.e., two or four filled channels). A CMOD of 0.1 mm was considered to represent a critical level of damage, particularly for samples cured for only 14 days, as the residual flexural strength at this stage is very low and most matrix ligaments have already been compromised. Exceeding this threshold would likely have caused the samples to fall apart. A 14-day healing period was selected based on previous studies indicating that this duration is sufficient for autogenous healing to take place. This timeframe also allows for a meaningful comparison with the effects of engineered healing processes activated by the tested healing agents.

Group 2 aimed to investigate the effect of sample age on healing performance. It is well-established that both autogenous healing and the chemical processes leading to C-S-H formation are generally more active at earlier ages [80]. The age at which the first crack was introduced was extended by 14 days more than in Group_1 (i.e. 28 days) while all other parameters were kept consistent with Group_1 (i.e., number of channels, CMOD of 0.1 mm, type of healing agents, and healing period).

The objective of Group_3 is to assess the impact of crack width reduction from 0.1 to 0.05 mm on both control samples and samples containing healing agents, tested after 14 or 28 days of curing. A crack width of 0.05 mm was barely visible to the naked eye and could be of the same order of magnitude as cracks caused by thermal shocks, such as shrinkage effects or freeze–thaw cycles. Moreover, the objective of Group 3 was to evaluate the medium-term healing performance of the tested systems over extended periods, specifically after 10 months (280 days) of curing, considering this a valuable extension to assess the system over time. This group aimed to investigate the durability and persistence of both autogenous healing (if any) and engineered healing processes.

Moreover, to assess the compatibility of nanolime dispersions as a healing agent in the NHL matrix and to determine whether the self-healing behaviour observed in hydraulic lime mortar samples is linked to the formation of calcium silicate hydrate (C-S-H), a key hydration product known for its binding and sealing properties in cementitious systems, complementary chemical and microstructural analyses were carried out on mock-up samples, as detailed in Sect. 2.1.2 and in accordance with the experimental plan outlined in Table 2.

**Table 2:** Experimental programme for material characterisation of mock-up samples

	Sample designation	Age of test (days)						Healing agent		Healing period (days)
		7	14	22	29	36	280	80:20	50:50	14
Mock-up samples	B2 + 7	✓	–	–	–	–	–	–	–	–
	B2 + 14	–	✓	–	–	–	–	–	–	–
	B2 + 22	–	–	✓	–	–	–	–	–	–
	B2 + 29	–	–	–	✓	–	–	–	–	–
	B2 + 36	–	–	–	–	✓	–	–	–	–
	C + 280^*^	–	–	–	–	–	✓	–		✓
	C + 280_80:20_H14^*^	–	–	–	–	–	✓	✓	–	✓
	C + 280_50:50_H14^*^	–	–	–	–	–	✓	–	✓	✓

^*^Samples collected from Group 3, as per Table 1

With respect to the mock-up samples (B2 + 7–36), two specimens were kept as untreated references, and their evolution over time was monitored using ATR FT-IR, XRD and SEM analyses, with measurements taken every 7 days up to 36 days of ageing.

To further investigate the effects of nanolime reactions on medium-term aged samples, three specimens were selected from vascular prismatic samples, specifically from Group 3 (i.e. 3_4_280_0.05). Two of the samples were treated with the tested nanolime dispersions (50:50 and 80:20) by fully immersing them in the solutions and maintaining magnetic stirring for 24 h at room temperature. The remaining specimen was retained as untreated references. After treatment, all samples were dried for 14 days under a constant nitrogen flux. This controlled atmosphere was selected to suppress carbonation and instead favour the formation of the C-S-H phase. In contrast, drying under ambient air conditions would likely have facilitated carbonation of the nanolime into calcium carbonate (CaCO₃), thus limiting the intended self-healing mechanism.

After the initial curing period, the samples were stored under controlled laboratory conditions (approximately 25 ± 5 °C and 50 ± 5% relative humidity) for 7, 28, or 280 days, depending on the specific requirements of each test. The following instrumentation and settings were used:

- ATR FT-IR Analysis was conducted using a *Thermo Nicolet™ NEXUS 870 FT-IR* spectrometer. Spectra were recorded in the 4000–650 cm^−1^ range, with a resolution of 2 cm^−1^, averaged over 128 scans.
- FT-IR in Transmittance Mode was performed with a *Shimadzu IRAffinity-1S FT-IR* spectrometer, collecting spectra in the 4000–400 cm^−1^ range.
- X-ray Diffraction (XRD) measurements were carried out using a *Bruker D8 “Da Vinci” diffractometer* with Cu-Kα radiation (λ = 1.5406 Å). Scans covered a 2θ range of 3° or 5° to 70°, with a step size of 0.03° or 0.05°, time per step of 0.3 s, at an operating voltage of 40 kV and current of 40 mA.
- Scanning Electron Microscopy (SEM observations were performed using a Zeiss Sigma FE-SEM. Images were acquired with a secondary electron (SE) detector at various magnifications, using an accelerating voltage of 10 kV. Small fragments (few millimetres size) were sampled with a scalpel and coated with a sputtered gold-layer.

## Experimental results

### Raw material characterisation

Firstly, ATR FT-IR and XRD analyses were carried out on sand and NHL 3.5 samples to identify their chemical and mineralogical compositions.

**Fig. 3:**
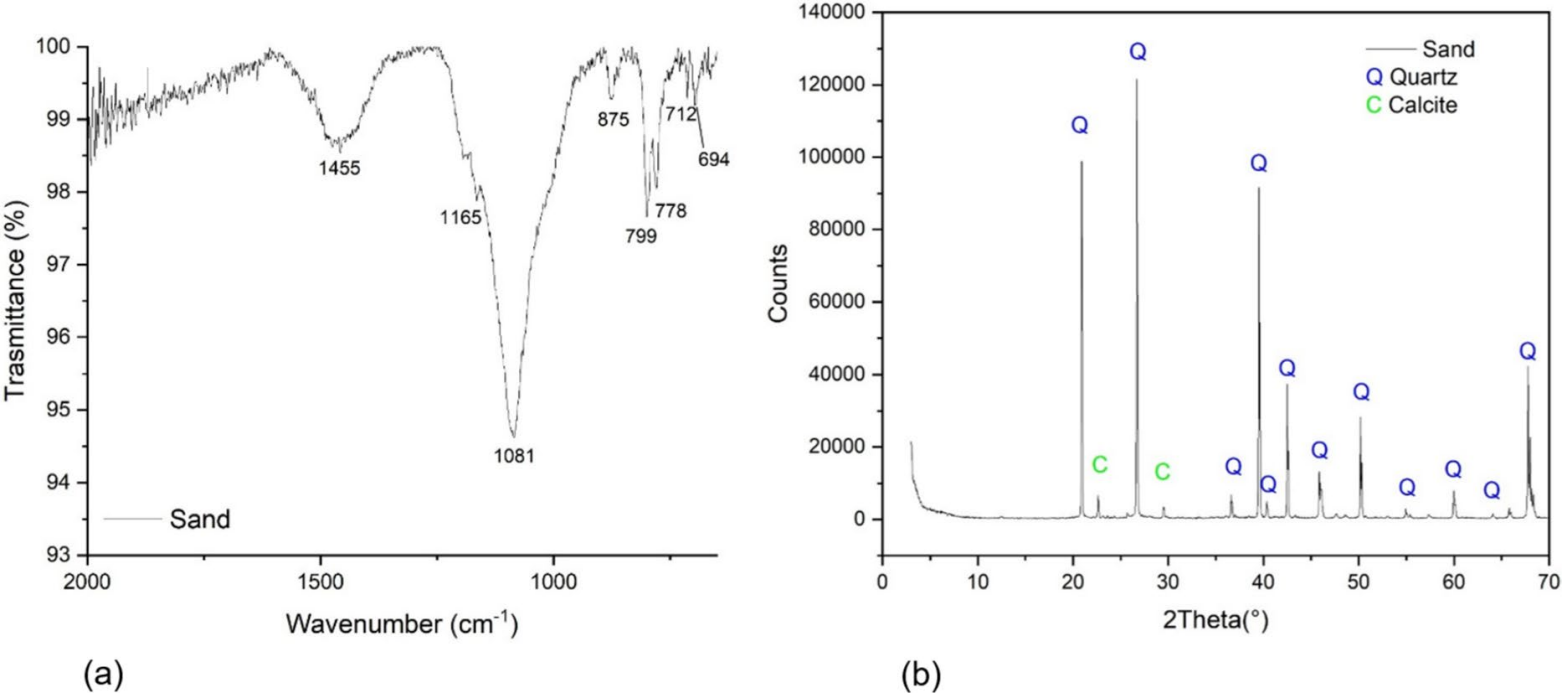
ATR FT-IR (**a**) and XRD (**b**) spectra of sand sample

Figure 3a shows the ATRFT-IR spectrum of sand, where characteristic absorption bands are observed at wavenumbers 1165, 1081, 799, 778 and 694 cm^−1^, corresponding to the vibrational modes of silica (Si-O-Si) indicating a dominant presence of quartz. Additional bands around 1455, 875 and 712 cm^−1^ are characteristic of calcite (CaCO₃). Figure 3b presents the XRD pattern of the same sand sample, indicating a dominant presence of quartz (Q) with sharp and intense diffraction peaks, labelled in blue, at 2θ angles of 20.9°, 26.7°, 36.6°, 39.5°, 40.3°, 42.5°, 45.8°, 50.2°, 54.9°, 60.0°, 64.1° and 67.9°. Minor peaks corresponding to calcite (C), marked in green, at 2θ angles of 22.9° and 29.5° are also visible, suggesting the presence of a small amount of calcium carbonate. Together, the FTIR and XRD analyses confirm that the sand is predominantly composed of quartz, with minor calcite impurities.

**Fig. 4:**
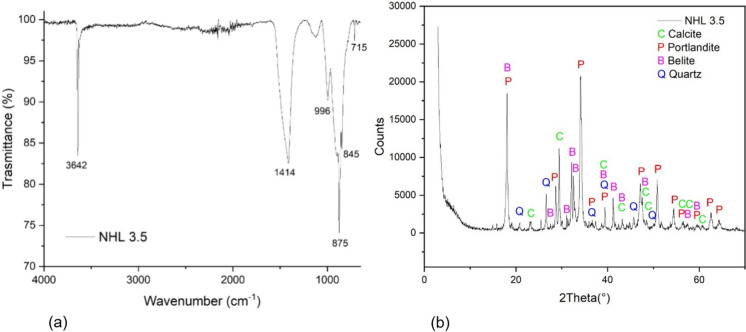
ATR FT-IR (**a**) and XRD (**b**) spectra of the hydraulic lime (NHL 3.5)

The hydraulic lime NHL 3.5 is composed of silicate phases, most probably of dicalcium silicate (Ca₂SiO₄, belite), calcite, and calcium hydroxide (portlandite), as shown in the ATR FT-IR spectrum Fig. 4a. The band at 3642 cm^−1^ corresponds to the O–H stretching vibration of portlandite (Ca(OH)₂), indicating the presence of calcium hydroxide. The broad and intense band at 1414 cm^−1^, along with the peaks at 875 cm^−1^ and 715 cm^−1^, are characteristic of calcite (CaCO₃), confirming the presence of carbonated phases. The bands observed at 996 cm^−1^ and 845 cm^−1^ are attributed to Si–O stretching and bending typical of silicate phases. These features are consistent with the presence of belite (*β-*Ca₂SiO₄), the probable main hydraulic component of the analysed lime; however, contributions from other silicate phases cannot be excluded [81].

The XRD spectrum Fig. 4b reinforces the findings from FT-IR analysis, displaying well-defined diffraction peaks associated with portlandite (P), calcite (C), and quartz (Q). The presence of silicate phases, most likely belite (Ca₂SiO₄), is supported by peaks at 2θ angles of 18.1°, 27.5°, 31.1°, 32.2°, 32.5°, 39.5°, 41.2°, 43.2°, 47.5°, 57.4°, and 59.5°; even though contributions from other crystalline phases cannot be excluded.

Portlandite (Ca(OH)₂) is identified by its characteristic reflections at 18.1°, 28.7°, 34.1°, 36.7°, 39.5°, 47.2°, 50.8°, 54.5°, 56.6°, 59.5°, 62.6°, and 64.1°. Peaks at 22.9°, 29.5°, 39.5°, 43.2°, 47.5°, 48.5°, 56.6°, 57.4°, and 60.7° correspond to calcite (CaCO₃), indicating the presence of carbonated phases. In addition, quartz (SiO₂) is detected by its distinct peaks at 20.9°, 26.7°, 36.6°, 39.5°, 45.8°, 50.2°, and 64.1°, likely originating from siliceous aggregates or natural impurities.

Together, the ATR FT-IR and XRD analyses indicate the likely presence of belite, portlandite, calcite, and minor quartz as main components of the hydraulic lime (NHL 3.5), nevertheless contributions from other phases cannot be excluded.

### Wettability results of healing agents

**Fig. 5:**
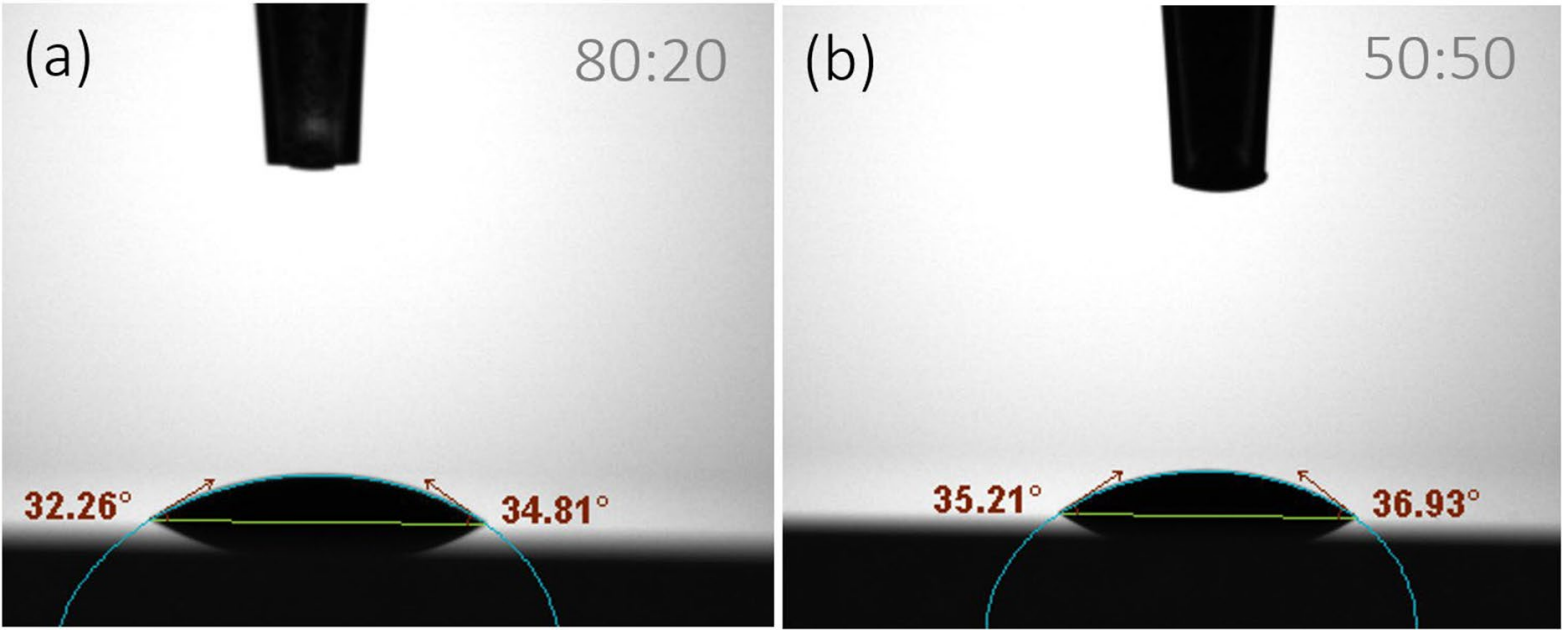
Healing agents, contact angle measurements: 50:50 nanolime dispersion (**a**), 80:20 nanolime dispersion (**b**)

The wettability results, presented in Table 3. The results represent the average of three measurements, with the coefficient of variation (CoV %) provided in brackets. Since all the healing agents tested are in aqueous solutions or dispersions, deionised water was also analysed as a reference to account for the influence of the solvent and ensure a reliable comparison of wettability characteristics. Representative contact angle measurements for each healing agent are shown in Fig. 5.

**Table 3:** Healing agents wettability results

Healing agents’ designation	Volume ml (CoV%)	Contact angle (θ_c_) deg (CoV%)		Baseline mm (CoV%)	
	0 s	0 s	10 s	0 s	10 s
Deionised water	5	22.09	22.07	4.29	4.24
50:50	3.37 (6)	37.78 (1)	36.28 (2)	3.60 (2)	3.59 (2)
80:20	5.07 (9)	35.84(5)	34.98 (5)	3.63 (16)	3.63 (15)

Regarding contact angle measurements, nanolime dispersions (50:50 and 80:20) exhibit significantly higher contact angles (37° and 36°, respectively) compared to deionised water (22°), indicating lower wettability. This is expected due to the presence of nanoparticles, which tend to increase surface roughness at the micro/nano scale and reduce the liquid’s ability to spread uniformly on the substrate. Although the two dispersions have similar contact angles, their spreading behaviour over time (i.e., Δqc over 10 s) differs: the 50:50 dispersion shows a larger decrease in contact angle (Δ*θ* ≈ − 1.50°), suggesting greater spreading, while the 80:20 dispersion maintains a more stable shape (Δθ*θ* ≈ − 0.86°). It is reasonable to assume that the higher ethanol content in the 50:50 dispersion makes it more prone to evaporation compared to the 80:20 dispersion. In terms of baseline measurements, both dispersions form more compact droplets (3.59–3.63 mm) than water (4.24–4.29 mm) with the 50:50 dispersion low CoV suggesting a more uniform surface interaction, whereas 80:20 has more variability. The 50:50 dispersion showed a lower applied volume (3.37 ml), likely due to the evaporation of the alcohol component.

### Mechanical test results

The results of the flexural tests conducted on prismatic natural hydraulic lime mortars containing either 2 or 4 channels -left empty or filled with a 50:50 dispersion (10 g/L calcium hydroxide in a 50:50 water–ethanol solution) or an 80:20 dispersion (10 g/L calcium hydroxide in an 80:20 water–ethanol solution), are presented and subdivided into Groups.

#### Group 1 results

The flexural test results of Group 1 are reported in Table 4 together with indices of healing derived from the load-CMOD responses. The results shown in Table 4 are the average of three specimens, with the coefficient of variation (CoV%) provided in brackets as an indicator of experimental variability.

**Table 4:** Group_1: flexural test results and indices of healing

	σ^k_14^_undamaged_ N/mm^2^ (CoV%)	σ^k_14^_damaged_ N/mm^2^ (CoV%)	σ^k_28^_healed_ N/mm^2^ (CoV%)	Healing indices	
				$\eta_{\sigma }^{k}$%	$\eta_{K}^{k}$ %
1_0_14	0.72 (1)	–		–	–
1_4_14_0.1	0.73 (16)	0.16 (24)	0.13 (18)	− 4	− 6
1_2_14_80:20_0.1	0.80(12)	0.19(36)	0.16(44)	− 5	− 3
1_2_14_50:50_0.1	0.80(0)	0.13(4)	0.11(6)	− 3	− 4
1_4_14_80:20_0.1	0.55(5)	0.10(18)	0.15(11)	12	12
1_4_14_50:50_0.1	0.54(2)	0.09(15)	0.14(7)	12	8

Firstly, it is worth noting that the Group 1 samples were tested at 14 days. The low cohesion and mechanical strength of the surface matrix hindered the secure attachment of the knife edges, which are essential for accurate CMOD measurement using clip gauges during flexural testing. In several cases, the knife edges detached along with portions of the surface mortar, indicating inadequate bonding. This may have occasionally contributed to variability in the data.

In Group_1, the presence of empty channels did not appear to affect mechanical performance, either in terms of strength or stiffness. Samples with empty channels, pre-cracked to a CMOD of 0.1 mm, exhibited no autogenous healing capability. Similarly, samples containing two channels filled with either 80:20 or 50:50 nanolime dispersion showed no noticeable healing effect.

However, when the volume of nanolime dispersion was doubled, by filling four channels, interesting healing behaviour was observed. In terms of healing efficiency, both the 50:50 and 80:20 nanolime dispersions resulted in a similar strength recovery of approximately 12%. Notably, the 80:20 mixture achieved a higher stiffness recovery of up to 12%, which is 4% greater than that of the 50:50 mixture.

#### Group 2 results

Prior to testing, all knife edges were carefully inspected to ensure they remained securely attached throughout the procedure. A 28-day curing period was deemed sufficient for adequate water evaporation and matrix hardening, allowing for the reliable application of knife edges and effective execution of the flexural tests. Only a few knife edges detached during the process, but these were promptly reattached before testing commenced.

Control samples tested to failure at 28 days, as well as those containing empty channels, exhibited flexural strengths of 0.97 N/mm^2^ and 0.99 N/mm^2^, respectively (Table 5: values represent the average of three specimens; CoV (%) is given in brackets.) Notably, over the following two weeks, a 26% increase in strength was observed in both sets of samples, regardless of the presence of channels. However, no recovery in strength or stiffness was detected in the samples with empty channels, confirming the absence of autogenous healing capacity within the matrix itself. Despite the inclusion of nanolime dispersion -regardless of the formulation used—no healing or strength recovery was observed whether two or four channels were filled. Considering the age of the samples (i.e. 28 days), it is reasonable to assume that the extent of damage required to induce a CMOD of 0.1 mm may have been too severe to allow for effective healing.

**Table 5:** Group_2: flexural test results and indices of healing

Group 2	σ^k_28^_undamaged_ N/mm^2^ (CoV%)	σ^k_28^_damaged_ N/mm^2^ (CoV%)	σ^k_42^_healed_ N/mm^2^ (CoV%)	Healing indices	
				$\eta_{\sigma }^{k}$	$\eta_{K}^{k}$
2_0_28	0.97(22)	–		–	–
2_4_28_0.1	0.99(27)	0.17(11)	0.14(14)	–4	–5
2_2_28_80:20_0.1	1.07(1)	0.13(3)	0.12(19)	–3	–3
2_2_28_50:50_0.1	1.07(6)	0.13(7)	0.10(15)	–1	–5
2_4_28_80:20_0.1	0.75(2)	0.17(13)	0.17(20)	0	–6
2_4_28_50:50_0.1	0.73(12)	0.16(17)	0.14(14)	–4	–4

#### Group 3 results

**Fig. 6:**
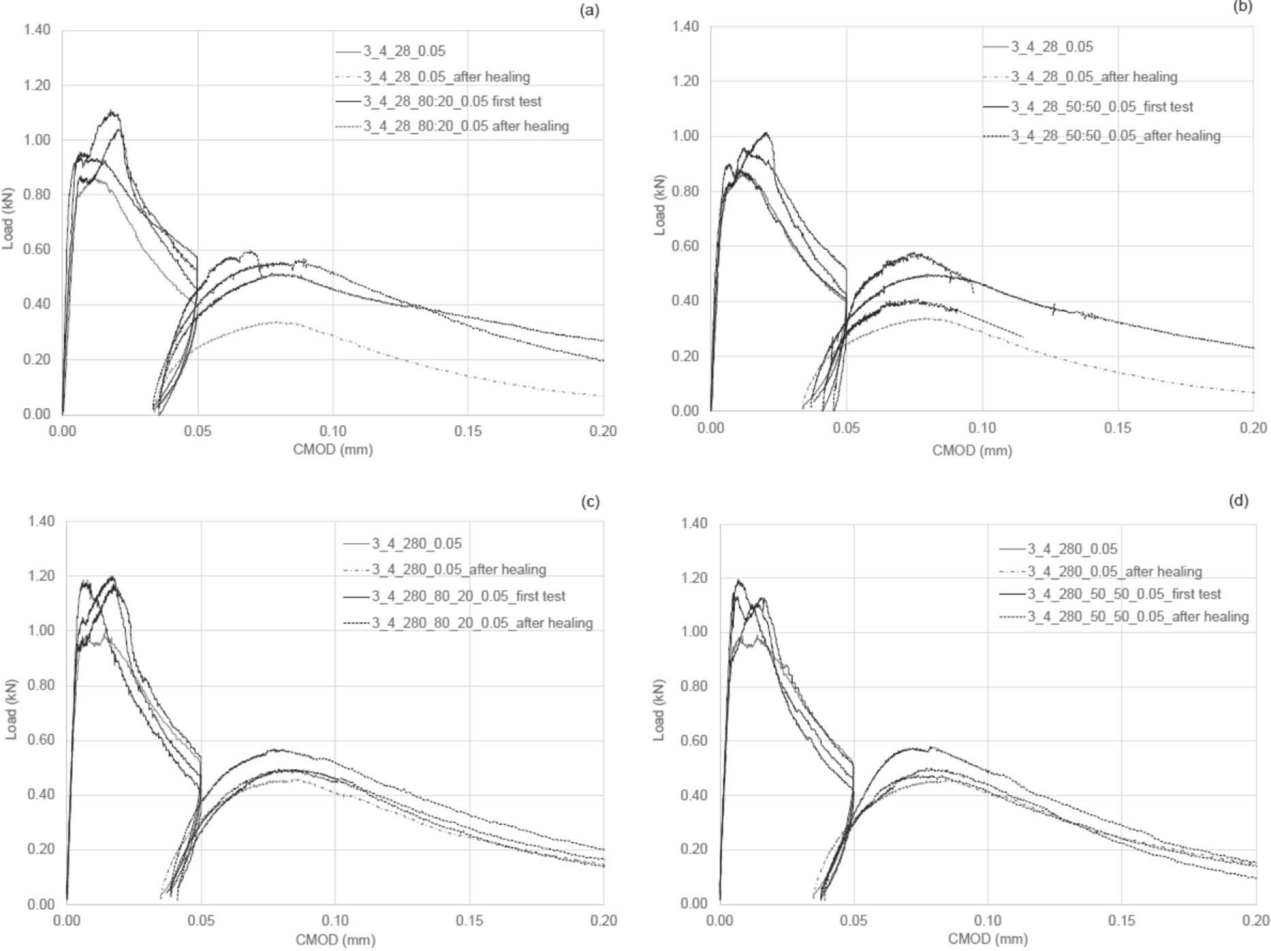
Mechanical response, Group_3. Samples tested at 28 days. Filled with 80:20 nanolime dispersion (**a**); filled with 50:50 nanolime dispersion (**b**). Samples tested at 280 days. Filled with 80:20 nanolime dispersion (**c**); filled with 50:50 nanolime dispersion (**d**)

In Group 3, the level of damage was reduced to a CMOD of 0.05 mm, while the age of the samples ranged from 28 to 280 days. All four channels were either left empty or filled with nanolime dispersions. The results of the flexural tests are reported in Table 6, mean values of three specimens; CoV (%) in brackets, and the Load versus CMOD graphs are shown in Fig. 6.

**Table 6:** Group_3: flexural test results and indices of healing

Group_3	σ^k_28^_undamaged_ N/mm^2^ (CoV%)	σ^k_28^_damaged_ N/mm^2^ (CoV%)	σ^k_42^_healed_ N/mm^2^ (CoV%)	Healing indices	
				$\eta_{\sigma }^{k}$	$\eta_{K}^{k}$
3_4_28_0.05	0.63(2)	0.27(4)	0.26(6)	–4	–1
3_4_28_80:20_0.05	0.73(7)	0.37(10)	0.40(5)	8	0
3_4_28_50:50_0.05	0.68(6)	0.32(10)	0.35 (14)	9	–2
3_4_280_0.05	0.7^*^	0.37^*^	0.33^*^	–13	–5
3_4_280_80:20_0.05	0.84(10)	0.34(10)	0.37(6)	6	–3
3_4_280_50:50_0.05	0.82(2)	0.33(8)	0.37 (8)	8	–2

^*^One sample was tested

**Fig. 7:**
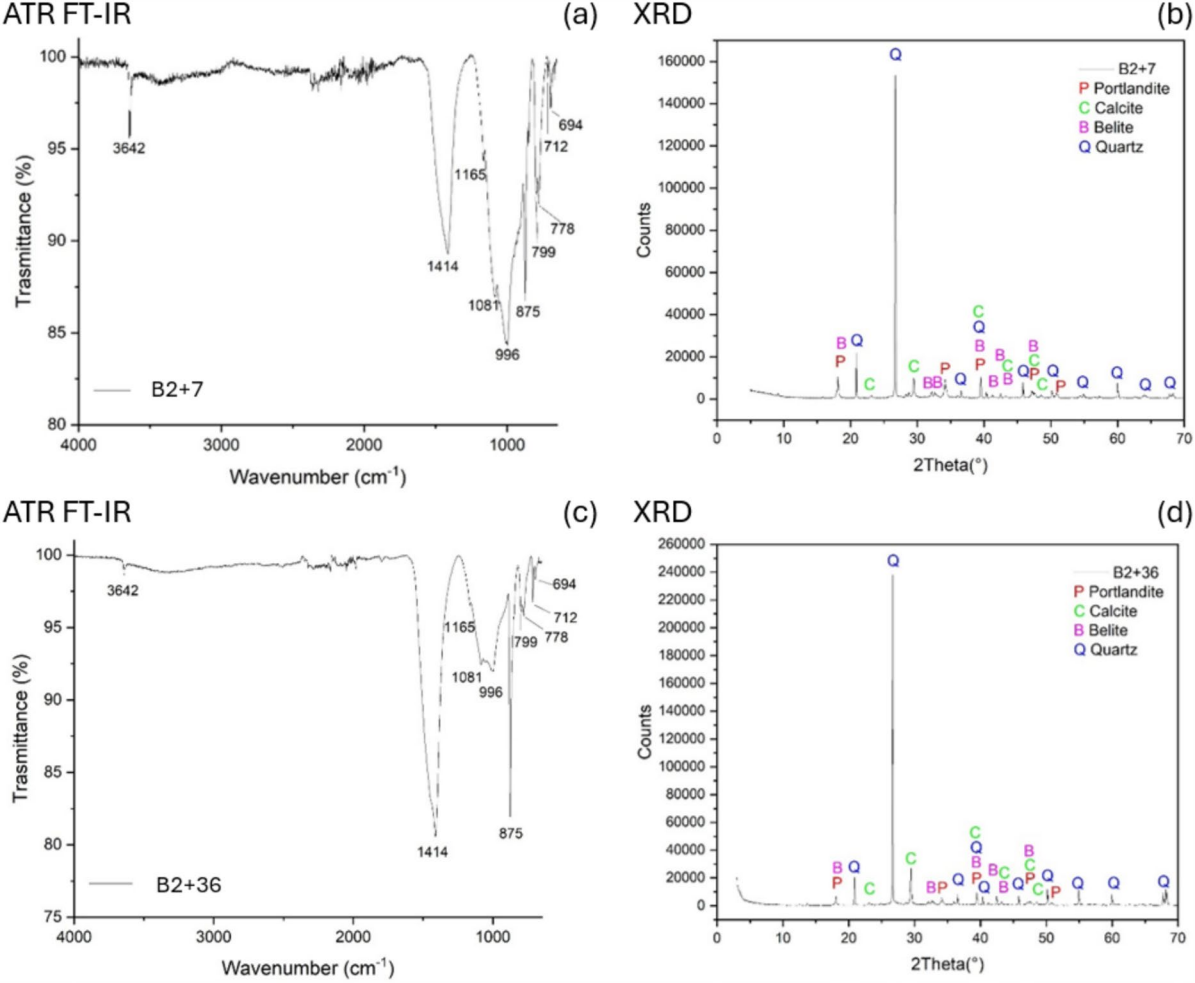
ATR FT-IR spectra of natural hydraulic lime mortar samples aged 7 and 36 days (**a**, **c**) and corresponding XRD patterns (**b**, **d**)

The reference samples with empty channels (i.e. 3_4_28_0.05) exhibited limited post-crack performance, with a reduction in peak flexural strength from 0.63 to 0.27 N/mm^2^ after damage, and no recovery (0.26 N/mm^2^) after 14 days of healing. This negative strength healing index (− 4%), suggests that NHL mortars show no autogenous healing capabilities in the absence of agents. In contrast, specimens filled with nanolime dispersions in ethanol–water solutions demonstrated enhanced performances. At 28 days, both the 80:20 and 50:50 dispersions led to higher initial strengths (0.73 and 0.68 N/mm^2^, respectively), with strength healing indexes of + 8% and + 9%, showing partial recovery of load-bearing capacity after crack formation. The stiffness-index recovery is neutral or slightly negative, suggesting the healed zone doesn’t fully restore stiffness. While the dispersion type did not drastically alter healing performance, a slightly higher ductility was observed for the 80:20 mixture (Fig. 7).

After extended curing to 280 days, both groups filled with nanolime dispersions exhibited slightly higher initial flexural strengths compared to their 28-day counterparts. In particular, the flexural strength increased by approximately 15% for the 80:20 dispersion and by about 21% for the 50:50 dispersion. Strength healing index at 280 days remained similar to the earlier stage: + 8% for the 50:50 dispersion and + 6% for the 80:20 dispersion. Both mixtures reached similar post-healing strengths of 0.37 N/mm^2^, indicating that extended curing time slightly enhanced healing but did not lead to substantial gains compared to the 28-day results. The stiffness-based recovery remained slightly negative in both cases.

The smooth, rounded post-peak shapes observed in the healed curves suggest a degree of crack bridging and possibly enhanced ductility introduced by the nanolime dispersions, which may contribute to a more gradual crack propagation, even if complete arrest of the crack is not achieved.

It is reasonable to assume that the nanolime dispersions contributed to a partial filling of microcracks, potentially lowering stress concentrations and enabling a more controlled evolution of damage. Among the two formulations, the 50:50 nanolime dispersion appears to offer slightly improved ductility compared to the 80:20 solution. This is evidenced by the shape of the healed load-CMOD curve, which shows a longer and more gradual post-peak descent, suggesting enhanced energy dissipation during fracture. The improved performance of the 50:50 dispersion may be attributed to the properties of its ethanol–water ratio carrier, which likely facilitates better transport and distribution of calcium hydroxide into fine cracks.

### Hydraulic lime mortar characterisation: FT-IR, XRD and SEM

This section presents the physicochemical characterisation of hydraulic lime mortars, focusing on the identification of hydration products and early reaction mechanisms observed within the initial 36-day curing period and up to 280**.**

#### Natural hydraulic lime mortars: reference samples aged 7–36 days

Representative ATR FT-IR and XRD spectra of mortar samples aged 7 and 36 days are presented in Fig. 7a, b and c, d respectively, illustrating the material behaviour over the observed ageing period.

In the ATR FT-IR spectra of both samples, the same components are detected. At 3642 cm^−1^, the characteristic O–H stretching band, associated with portlandite (Ca(OH)₂), is present. The bands at 1414, 875, and 712 cm^−1^ are indicative of calcite (CaCO₃). The bands at 1165, 1081, 799, 778 and 694 cm^−1^ are associated with quartz. Additionally, bands at 996 and 845 cm^−1^—compatible with the presence of belite (Ca₂SiO₄)—are visible in both spectra, suggesting the persistent presence of unreacted or slowly hydrating hydraulic phases. These results are in line with previous studies, which attribute this behaviour to the composition of the hydraulic lime. Indeed, the likely presence of belite (Ca₂SiO₄, or C2S in cement chemistry), a compound known for its slow reaction kinetics, could explain the slow hydration process of the silicate phases; in contrast with conventional concrete typically containing alite (Ca₃SiO₅, or C3S), which reacts more rapidly and forms calcium silicate hydrate (C-S-H) within approximately 28 days [82].

The XRD patterns support this interpretation. For both 7 and 36-days samples, peaks for portlandite (P), calcite (C), belite (B), and quartz (Q) are observed. Belite reflections remain visible, consistent with the slow hydration kinetics of this phase.

Importantly, no clear evidence of calcium silicate hydrate (C-S-H) formation is observed in either the ATR FT-IR or XRD data. This suggests that C-S-H has not yet formed in detectable quantities by day 36, aligning with the known slow hydration behaviour of hydraulic lime mortars, especially under ambient curing conditions [82].

#### Natural hydraulic lime mortars: reference and treated samples aged 280 days

As discussed in Sect. 3.4.1, natural hydraulic lime containing belite (Ca₂SiO₄, or C2S in cement chemistry) exhibits slow reaction kinetics. In the early stages of curing, up to 36 days, no calcium silicate hydrate (C-S-H) typically forms. Therefore, the present section explores the medium-term development of these mortars, focusing on mineralogical and microstructural transformations observed over a 10-month period (280 days). The analysis aims to evaluate the progression of hydration and carbonation, along with the potential role of nanolime dispersions in promoting the formation of binding phases such as C-S-H and related compounds.

**Fig. 8:**
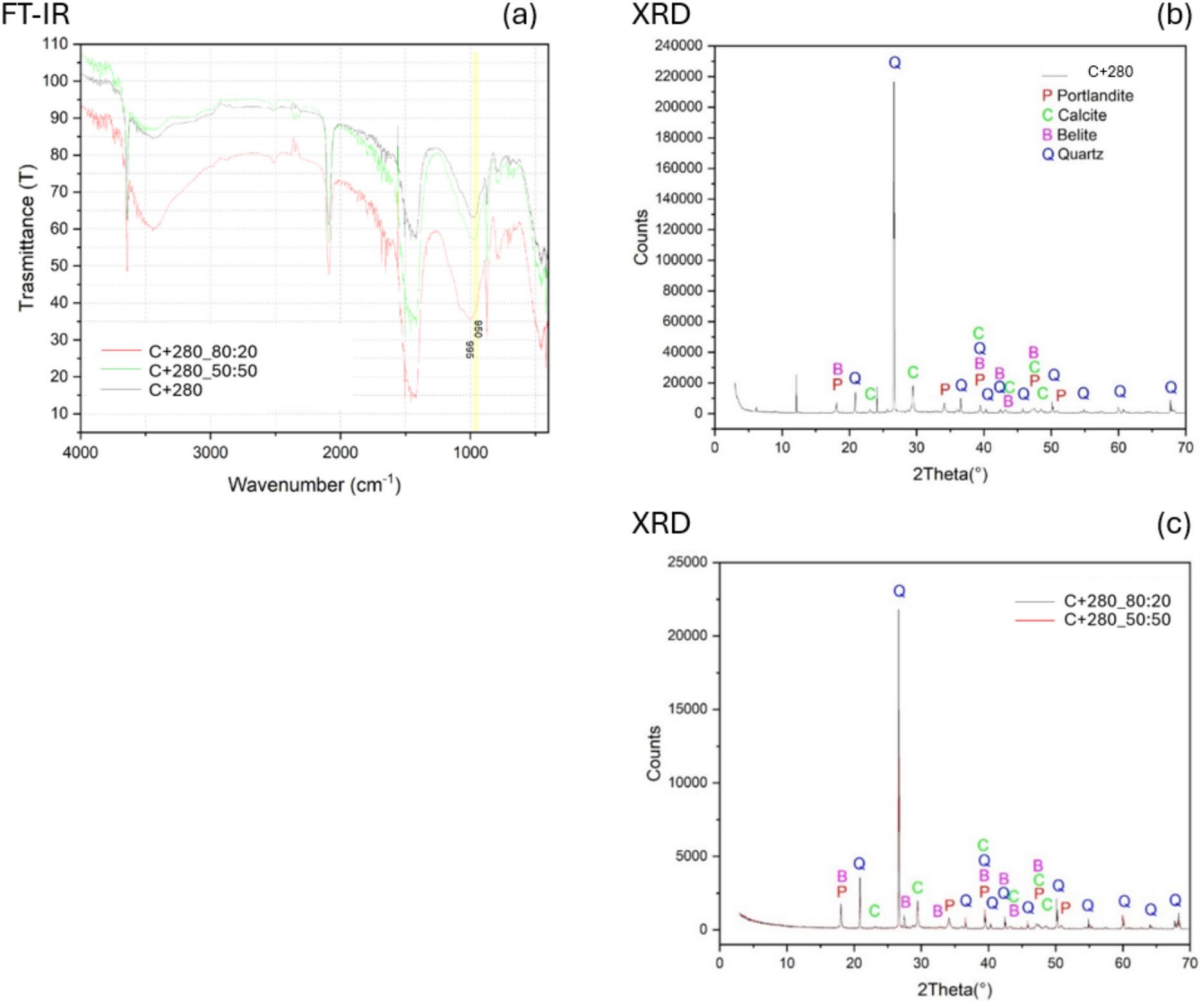
FT-IR and XRD spectra of natural hydraulic lime mortar aged 10 months: untreated (**a**, **b**); treated with nanolime dispersions (**a**, **c**)

Figure 8 presents the FT-IR, in transmittance mode, and XRD spectra of natural hydraulic lime (NHL) mortar samples aged 10 months, both untreated and treated with nanolime dispersions. Firstly, the untreated mortar was characterized by FT-IR (Fig. 8a, black line). The spectrum shows dominant absorption bands at ~ 1414, 875, and 712 cm^−1^, characteristic of calcite. Moreover, a band at ~ 3642 cm^−1^ suggests the presence of portlandite. Interestingly, a broad band near ~ 996 cm^−1^, likely belite, can be observed, along with a weak shoulder around 950 cm^−1^ (highlighted by a yellow stripe) that is characteristic of C-S-H [83]. Therefore, the hydraulic lime mortar samples, after 10 months of curing show that calcium silicate hydrate (C-S-H) is formed. In Fig. 8a, comparing the nanolime-treated samples (C + 280_80:20 red line and C + 280 _50:50 green line), the overall phase composition remains consistent with the untreated sample and the weak shoulder of C-S-H around 950 cm^−1^ remains present in both samples. The comparison of the treated samples, with both the nanolime formulations, with the untreated sample is complex because the C-S-H characteristic band is not sharp and well-defined, but it is a weak shoulder. Therefore, it is complicated to evaluate the variation of the relative ratios between the belite and the C-S-H band to tell if the formation of the latter is due to the nanolime treatment or to the natural curing of the mortar.

The X-ray diffraction (XRD) patterns presented in Fig. 8b, c provide insight into the crystalline phases present in the untreated (C + 280) and nanolime-treated mortar samples (C + 280_80:20 and C + 280_50.50). In Fig. 8b, the dominant reflections correspond to quartz (Q), portlandite (P), calcite (C), and belite (B), indicating the presence of not completely reacted raw materials, as belite, that still after 10 months is not completely reacted to form C-S-H. No identifiable peaks for C-S-H were detected in the XRD pattern, probably due to its amorphous nature [84] and the complexity of the mortar matrix. In Fig. 8c, comparing the nanolime-treated samples (C + 280_80:20 and C + 280_50:50), the overall phase composition remains consistent with the untreated sample. In these samples, as well, no identifiable peaks for C-S-H were detected in the XRD pattern reinforcing the interpretation of its amorphous nature.

In conclusion, it is worth noting that the detection of C-S-H (calcium silicate hydrate) in FT-IR and XRD analysis of natural hydraulic lime (NHL) systems presents inherent challenges. In FT-IR analysis, the Si–O asymmetric stretching bands characteristic of C-S-H are not well-resolved, as often partially overlap with the more intense absorption bands of other silicate phases, further obscuring its detection. Regarding XRD, the C-S-H is a poorly crystalline or amorphous phase, lacking the long-range atomic order required to produce distinct diffraction peaks. Instead, it appears as broad, weak humps in the XRD pattern that are easily overshadowed by sharper signals from more crystalline phases such as portlandite, calcite, and quartz.

#### SEM morphological analysis

Morphological analysis using SEM was conducted on both untreated and nanolime-treated samples after 280 days of curing, as shown in Fig. 11, to identify reaction products and provide insight into potential self-healing mechanisms.

**Fig. 9:**
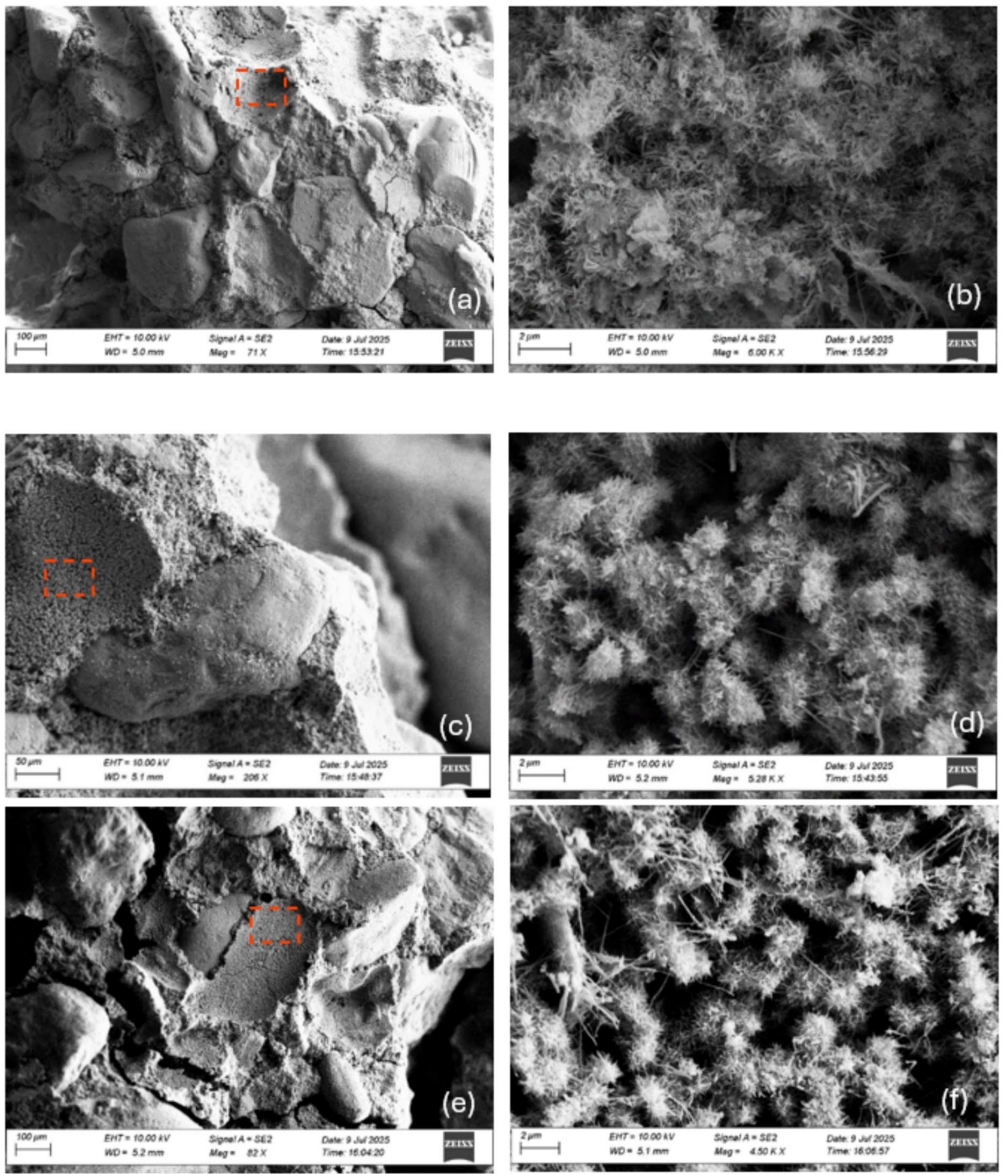
SEM images of natural hydraulic lime mortar samples aged 280 days: reference sample C + 280 (**a**, **b**); sample treated with 50:50 nanolime dispersion, C + 280_50:50 (**c**, **d**); and sample treated with 80:20 nanolime dispersion, C + 280_80:20 (**e**, **f**)

Figure 9a shows the reference natural hydraulic lime mortar sample (C + 280) at a relatively low magnification (71 ×). The matrix appears composed of coarse mineral particles, likely the quartz sand grains, embedded in a finer binder phase. At higher magnification (600 k ×), the reference matrix (Fig. 11b) reveals a surface populated by flock-like or fibrous crystalline formations, which are associated with calcium-silicate-hydrate (C-S-H) late-stage hydration products, that are well connected to each other [85].

Figure 11c, d display the mortar sample treated with a 50:50 dispersion (C + 280_50:50), while Fig. 11e, f show the one treated with an 80:20 dispersion (C + 280_80:20). At lower magnification, the matrix appears somewhat slightly more consolidated and homogeneous compared to the reference sample shown in Fig. 11a. At higher magnification, the matrix reveals similarly organised network of fibrous or flocks-like crystals, corresponding to calcium-silicate-hydrate (C-S-H), as in Fig. 9b.

The overall microstructural features remain remarkably similar across both treated and untreated mortars. This lack of visually distinguishable differences at the microscale is an important indication of material compatibility. The fact that nanolime treatments do not disrupt the inherent microstructural characteristics of the natural hydraulic lime matrix supports the conclusion that the intervention is chemically and physically compatible with the original material.

#### Perspective and limitations

The results presented in this study contribute to the growing body of research on engineered self-healing strategies for lime-based mortars and provide new insight into the specific role of nanolime dispersions when delivered through vascular systems. From a broader perspective, the findings confirm that nanolime can act as a compatible and durable healing agent for natural hydraulic lime mortars, particularly within crack-width regimes that are most relevant for historic masonry. The persistence of healing activity up to 280 days highlights the potential of nanolime-based systems to support long-term crack stabilisation. This aligns well with conservation principles that prioritise durability, compatibility, and minimal intervention.

At the same time, the measured healing efficiencies remain moderate, with strength recovery typically limited to the range of 8–12% and negligible stiffness recovery. This indicates that the primary contribution of nanolime dispersions lies in partial crack filling, stress redistribution, and crack-bridging effects, rather than in the full recovery of the original mechanical properties. Within a heritage conservation framework, this behaviour is consistent with compatible intervention strategies that aim to control damage evolution and limit exposure to aggressive environmental agents. Fine cracks are of particular concern in historic masonry, as their progressive development under sustained or repeated actions may contribute to degradation and increased structural vulnerability, even in the absence of clear visual indicators [86, 87]. In this context, the stabilisation of micro-cracking and the redistribution of stresses, even if partial, might contribute meaningfully to the long-term durability and safety of masonry structures.

Several limitations of the present study should be acknowledged. First, the investigation was carried out under controlled laboratory conditions, with constant temperature and relative humidity, and without exposure to cyclic environmental actions such as wetting–drying, freeze–thaw, or salt crystallisation. These factors are known to strongly influence both autogenous and engineered healing mechanisms in lime-based materials. Secondly, the experimental setup was intentionally simplified to ensure repeatability and enable direct comparison between specimens, allowing the specific contribution of the healing agent to be assessed under controlled conditions. As a result, the testing configuration does not fully capture the complexity of real damage scenarios in historic masonry. Second, the investigation focused on controlled crack widths of 0.05 and 0.1 mm, while larger cracks and recurrent damage were not considered. The study also adopted a simplified flexural configuration with a single activation of the vascular system and direct agent injection, without addressing the additional complexities or potential benefits of fully integrated, automated, or repeatedly activated vascular networks. Third, although FT-IR, XRD, and SEM analyses suggest morphological and chemical compatibility between nanolime treatments and the NHL matrix, the identification and quantification of newly formed C-S-H phases remain challenging. The amorphous nature of C-S-H and its overlap with signals from existing hydraulic phases limit the ability to conclusively distinguish treatment-induced products from those formed through natural long-term hydration.

Further research should explore alternative vascular designs, such as branched, interconnected, or 4D-printed networks, to enhance delivery efficiency and long-term performance in heritage masonry. In practical conservation applications, such advancements could enable the integration of this approach within lime-based repair mortars or repointing interventions, offering a compatible strategy to mitigate micro-cracking and limit moisture ingress over time.

## Conclusion

This study investigates the use of nanolime dispersions as healing agents in natural hydraulic lime mortar matrices, tested over a period from 14 to 280 days, and considering a 14-day healing period. Sacrificial channels were embedded into prismatic samples to allow direct interaction between the healing agent and the binder matrix, isolating the agent’s specific contribution to the overall healing performance. Two nanolime dispersions (10 g/L calcium hydroxide) were prepared with different water–ethanol ratios: 50:50 and 80:20. Based on the analyses presented, the main conclusions are as follows:

- Nanolime dispersions showed promise as healing agents for self-healing lime-based mortars, making them especially relevant for heritage conservation. Healing effectiveness was influenced by both sample age and crack width. At 14 days, only samples with four filled channels showed recovery, reaching ~ 12% strength regain, with 80:20 slightly outperforming 50:50 in stiffness. At 28 days, no healing occurred in samples with larger (0.1 mm) cracks, regardless of formulation or dosage. However, reducing the crack width to 0.05 mm enabled recovery of up to 9% (50:50) and 8% (80:20). At 280 days, similar healing performance was maintained, confirming the long-term activity of nanolime, though stiffness recovery remained negligible.
- Nanolime dispersions likely contribute to healing by partially filling microcracks. The post-peak curves suggest a degree of crack bridging and possibly enhanced ductility introduced by the nanolime, which may lead to more gradual crack propagation, even if complete crack arrest is not achieved.
- Through FT-IR and XRD analyses, the detection of C-S-H remains challenging due to overlapping signals from other compounds and to its amorphous nature. It is also worth noticing that the extreme compatibility of the C-S-H phase, that naturally forms in hydraulic lime mortars, further complicates the distinction of the treatment induced C-S-H formation. Although clear evidence of C-S-H formation was limited, the increase of healing performance in the mechanical data suggested possible hydration reactions potentially enhanced by nanolime.
- SEM analyses showed subtle morphological improvements in the treated samples, such as fewer visible interfacial defects between matrix and aggregates. Needle-like or fibrous crystalline formations, likely associated with C-S-H, were observed in both treated and untreated mortars, and the lack of clear distinction indicates a high level of compatibility.
